# Supplementary material for: Self-Assessment of Preparedness among Critical Care Trainees Transitioning from Fellowship to Practice
Source: Healthcare (Basel). 2019 May 30;7(2):74. doi: 10.3390/healthcare7020074 (PMC6628175; doi:10.3390/healthcare7020074)
Supplement: Supplementary file 1 [file healthcare-07-00074-s001.pdf]

healthcare

Supplemental Materials

In-Training Section Research Survey

* Required

1. 1. What is your current position: *

Mark only one oval.

- Healthcare Non-Physician In training
- Recent Healthcare Non-Physician Graduate (1-3 years)
- Physician In Training (Clinical Fellowship, Residency, Research Fellowship)
- Physician who graduated training within the past 1-3 years (please choose most advanced training)
- Other: ____________________

2. 2. What is your specialty (please include all that apply)? *

Check all that apply.

- Critical Care Internal Medicine
- Critical Care Pediatrics
- Critical Care Neurology
- Critical Care Surgery
- Anesthesia– Critical Care
- Other: ____________________

3. 3. Have you in the past 4 years interviewed or are you currently interviewing for a job? *

Mark only one oval.

- Yes
- No

4. 4. What is the location of interest (if interested in more than one please include all that apply)? *

Check all that apply.

- ☐ Northeast
- ☐ Southeast
- ☐ Northwest
- ☐ Southwest
- ☐ Northcentral
- ☐ Southcentral
- ☐ Anywhere in US
- ☐ Other: ____________________

5. 5. What is your job preference? *

Mark only one oval.

- ◯ Full time academic
- ◯ Full time private practice
- ◯ Part time academic
- ◯ Part time private practice
- ◯ Other: ____________________

6. 6. If you have interviewed in the past 4 years, or have already scheduled your interviews, how many locations will you interview? Please offer a number otherwise please skip the question. *

7. 7. If you interviewed in the past 4 years or will interview soon, what did you use/ or do you intend to use as a source of available jobs (please choose all that apply): *

Check all that apply.

- ☐ SCCM site
- ☐ Direct contact (phone, email, letter) with potential employers Head hunter/recruiter
- ☐ Word of mouth
- ☐ Suggestions offered by my mentor/program director
- ☐ N/A
- ☐ Other: ____________________

8.8. The next questions (8–14) will be evaluated based on a Likert scale. If you interviewed in the past 4 years or will interview soon please choose the best option (otherwise please choose N/A): *

Mark only one oval per row.

	I strongly agree	I agree	I neither agree nor disagree	I disagree	I strongly disagree	N/A
8. I felt/feel well prepared for the interviewing process:	⬭	⬭	⬭	⬭	⬭	⬭
9. I felt /feel well prepared about creating an adequate portfolio for job interviews (building a CV, obtaining appropriate letters of recommendation).	⬭	⬭	⬭	⬭	⬭	⬭
10. I received formal guidance from my mentor/training program in preparing my portfolio.	⬭	⬭	⬭	⬭	⬭	⬭
11. I received guidance or help from mentor mentor/training program in searching for a position.	⬭	⬭	⬭	⬭	⬭	⬭
12. I received guidance or help from my mentor/training program in deciding what position to choose.	⬭	⬭	⬭	⬭	⬭	⬭
13. My training program facilitated my resume building throughout my training period.	⬭	⬭	⬭	⬭	⬭	⬭
14. I consider a formal training course in job search, portfolio development and details of the interview process very useful.	⬭	⬭	⬭	⬭	⬭	⬭

9. **15. If you agree it is important to participate in a formal course in job search, portfolio development and details of the interview process, please choose all that interest you from all the following categories:**

Check all that apply.

- [ ] Course offered by home institution
- [ ] Course at SCCM Annual Congress
- [ ] Webinar

10. **16. If you agree it is important to participate in a formal course in job search, portfolio development and details of the interview process, please indicate when this is best offered:**

Check all that apply.

- [ ] At the beginning of training
- [ ] In the middle of training
- [ ] Towards the end of training
- [ ] Throughout training
- [ ] Independent of training

11. **17. Please choose from the following topics the ones you find most interesting and helpful for a formal course in job search, portfolio development and details of the interview process (please choose all that apply):**

Check all that apply.

- [ ] Understand career pathways after Critical Care Training
- [ ] Mock job Interview
- [ ] Work Life Balance
- [ ] How to obtain a dream job.
- [ ] Post interview, when is the right time to start to negotiate, and how?
- [ ] Academia vs. private practice: what is similar, what is different?
- [ ] How to build a CV and develop a portfolio.
- [ ] How to find mentorship in a new job.
- [ ] Signing the contract - money, insurance, liability, taxes

12. **18. Please choose from the following topics the ones you find most interesting as other possible topics that could be offered at SCCM Annual Congress or via Webinars:**

Check all that apply.

- [ ] Academic tenure and promotion
- [ ] How to obtain funding in the current era
- [ ] Tips for improving coding and billing
- [ ] Dealing with being sued
- [ ] Constructive communication and managing conflict
- [ ] Leadership vs. facilitative situational leadership

13. **Please add any suggestions in the following Free Textbox. Thank you very much for your participation!**
